# From the nucleus to the apoplast: building the plant’s cell wall

**DOI:** 10.1093/jxb/erv522

**Published:** 2015-12-29

**Authors:** Nadav Sorek, Simon Turner

**Affiliations:** University of California, Berkeley; University of Manchester, UK

The plant cell wall has a crucial role in any aspect of a plant’s life cycle and, as a result, cell wall research touches all aspects of plant biology (Keegstra, 2010). New and exciting work on cell wall research is constantly being published that has refined and sometimes even challenged the way we see cell wall biosynthesis and functions. The goal of this special issue is to provide reviews that will present our current thinking on different aspects of plant cell wall research.

J Bidhendi and Geitman (2016) review the area of the plant cell wall and morphogenesis. Plant expansion relies on the balance between turgor pressure and the elasticity of the cell wall (Smolarkiewicz and Dhonukshe, 2013); consequently, cell wall remodelling is essential for plant morphogenesis (Yanagisawa *et al.*, 2015). In their review, Jafari Bidhendi and Geitman describe how the structure and modification of cell wall polymers affect the mechanical properties of the cell wall. They present very interesting thoughts on pectin structure and how pectin modification contributes to the mechanical properties of the cell wall.

With a direct link to the cell wall and morphogenesis, Daniel Cosgrove (2016) describes what defines cell wall extensibility. Cosgrove re-examines the implementation of Young’s modulus of elasticity on the plant cell wall (McKee *et al.*, 2011), and describes the caveats we should be aware of when applying Young’s modulus of elasticity to the plant cell wall. Cosgrove continues by examining the factors that contribute to cell wall loosening, with an emphasis on the interaction between the different cell wall polymers. The review finishes with an artistic representation of a model of the cell wall.

Borassi *et al.* (2016) describe the connection between the cell surface and the cell wall, with an emphasis on extensin. Extensins can self-assemble into a well-defined network (Cannon *et al.*, 2008), however, their exact role is far from being resolved (Lamport *et al.*, 2011). Borassi and colleagues provide a thorough description of the divisions to subclasses present within the extensin family. The authors not only describe the typical motifs for each class, but also connect these to the phenotypes of known extensin mutants and present our current knowledge on the extension function. Extensins that possess a kinase or formin domain may act in signalling while other extensins, with a more classic or typical structure, contribute to cell wall architecture.

A particular example of signalling at the cell surface continuum is discussed by Bruce Kohorn (2016), who has focused on wall-associated kinases. Wall-associated kinases (WAKs) bind largely to the pectin fractions but may also bind other substrates such as glycine-rich proteins (Park *et al.*, 2001). WAKs possess a cytoplasmic kinase domain that can transduce cell wall architecture information to the intracellular signal transduction pathways allowing WAKs to act as cell wall sensors. Different pectin fragments bind WAKs with different affinities and initiate separate signal cascades. The result is that WAKs are able to differentiate changes in the cell wall caused by cell expansion from those caused by biotic or abiotic stress.

Staying with pectin, Charles Anderson (2016) discusses our current knowledge on pectin biosynthesis. Anderson describes the knowledge gap we have between pectin structure and pectin biosynthesis. Live imaging during the course of hypocotyl expansion has shown that the cellulose synthase complex is inserted into the plasma membrane thousands of times (Paredez *et al.*, 2006). A similar rate of pectin/hemicellulose deposition would require massive trafficking of cell wall material containing vesicles. Anderson describes some of the emerging tools to monitor pectin biosynthesis in live cells that can advance our knowledge on how cell wall polymer deposition is co-ordinated.

Wang and Hong (2016) review the recent advance in using solid-state NMR (SSNMR) to look at the 3D architecture of plant cell walls. The use of SSNMR to investigate spatial proximities and structures revealed new insights into the arrangement of the cell wall polymers (Dick-Perez *et al.*, 2011, 2012; Dupree *et al.*, 2015). One of the key findings is the abundance of interactions between cellulose, hemicellulose, and pectin (Dick-Perez *et al.*, 2011, 2012). New data imply that there are few interactions between cellulose and hemicellulose and that cellulose fibrils are not ‘coated’ by hemicellulose. Instead, there are many cellulose–pectin interactions suggesting that the primary cell wall is a tangled network of polymers. Wang and Hong (2016) also used SSNMR to investigate the properties of the cellulose fibres and, more specifically, the number of glucan chains in an individual cellulose fibre. Their data suggest that cellulose microfibrils in plant primary walls must be sufficiently large to contain at least 24 chains.

Moving from the primary cell wall, Kumar *et al.* (2016) provide a comprehensive review on secondary cell walls. The lack of pectin and the abundance of different hemicelluloses (e.g. xylans and mannans) and lignin, gives secondary cell walls completely different properties from primary cell walls (Vogel, 2008). In recent years, there have been multiple breakthroughs in the field, from the large-scale identification of transcription factors (Taylor-Teeples *et al.*, 2015) to the biosynthesis of lignin (Bonawitz *et al.*, 2014) as well as the visualization of cellulose biosynthesis in secondary walls (Watanabe *et al.*, 2015). The authors describe all aspects of secondary cell walls, from transcription regulation to polymer biosynthesis and structure with a focus on the secondary cell wall.

Tateno *et al.* (2016) describe the use of cellulose biosynthesis inhibitors (CBIs) to study cellulose synthesis. The best characterized CBI is isoxaben, which causes the clearance of the cellulose synthase complex from the plasma membrane (DeBolt *et al.*, 2007). Interestingly, mutants which are resistance to isoxaben (*ixr* mutants) seem to be specific to the CESA family (Scheible *et al.*, 2001). In recent years, several more CBIs have been identified (Brabham *et al.*, 2014; Xia *et al.*, 2014). The authors describe the different CBIs and how they can be categorized into three groups, CBIs that clear CESA particles from the plasma membrane (e.g. isoxaben), CBIs that stop CESA movement at the plasma membrane (e.g. DCB), and CBIs that alter the CESA trajectory at the plasma membrane (e.g. Morlin). An increasing number of CBIs, with their distinct modes of action are already being used and their use probably will become even more prevalent in studies aimed at investigating cellulose biosynthesis.

From an environmental perspective, Wang *et al.* (2016) discuss how abiotic stresses affect cellulose synthesis, an emerging area in the field of cellulose synthesis (Endler *et al.*, 2015). The connection between the cellulose synthase complex and microtubules is well established (Bashline *et al.*, 2014), as well as accessory proteins in cellulose biosynthesis (McFarlane *et al.*, 2014). The authors nicely connect environmental cues, the machinery that surrounds cellulose biosynthesis and regulators, such as abscisic acid. Another interesting point raised by the authors concerns the cellulose synthase complex itself. There is evidence that the complex is being phosphorylated (Chen *et al.*, 2010), however, it is not clear how phosphorylation affects the complex, and if and how phosphorylation regulates cellulose synthesis in response to environmental cues.

To summarize, cell wall research touches many disciplines, starting with environmental cues, to transcription regulation, vesicle trafficking, and polymer biosynthesis and deposition. Hence the title of this special issue: from the nucleus to the apoplast: building the plant’s cell wall. We thank all the authors for their interesting reviews, and all the reviewers for their contribution. We hope you will enjoy reading articles in this issue.
